# A cross-sectional study of determinants of birth weight of neonates in the Greater Accra region of Ghana

**DOI:** 10.1186/s40748-015-0023-4

**Published:** 2015-10-07

**Authors:** Margaret Atuahene, David Mensah, Martin Adjuik

**Affiliations:** School of Public Health, University of Ghana, Legon, Accra, Ghana; National Malaria Control Programme, Accra, Ghana; INDEPTH Network Secretariat, Accra, Ghana

**Keywords:** Birth weight, Anthropometric measurements, Neonates, Low birth weight, Normal birth weight

## Abstract

**Background:**

Birth weight is a major determinant of infant morbidity and mortality. Fetal undernourishment means an increased risk of dying during a baby’s early months and years. Birth weight has emerged as the leading indicator of infant health and welfare and the central focus of infant health policy. The issues have not been comprehensively evaluated in part due to lack of or limited empirical data. To this end, this study is aimed to evaluate the effects of maternal determinants on the birth weights of neonates in two major hospitals.

**Results:**

Low birth weight neonates were significantly (*p* < 0.001) associated with low gestation at birth (34.8 ± 3.8) while mothers of low birth weight neonates had significantly (*p* = 0.034) lower body mass index (27.3 ± 5.4) than their normal birth weight counterparts (29.0 ± 6.3). Gestation at birth (*p* < 0.001), diastolic blood pressure (*p* = 0.008) was the only significant determinant of birth weight.

**Conclusion:**

An increase in gestation at birth by 1 week results in over twice more likelihood of a normal birth weight while a rise in diastolic blood pressure is less likely to give rise to a normal birth weight neonate.

## Background

Low birth weight (infants born weighing less than 2500 g) is a very important phenomenon with respect to birth outcomes in the developing world [1]. Birth weight is a major determinant of infant morbidity and mortality [2, 3]. Existing literature has established the associations between LBW and infant mortality [4]. This is a major reason that attention to determinants of LBW has to be given much priority in public health.

Many studies have been conducted all over the world and a wide range of risk factors for LBW recorded over the years [5]. These range from maternal demographic and maternal anthropometric to maternal nutrition. Among the demographic risk factors are young maternal age [6], low education [7] and primiparity [8].

A major determinant of birth outcomes is the nutritional status of mothers during pregnancy [9]. Other indicators that have shown associations with birth outcomes happen to be maternal pre pregnancy body mass index and weight gain during pregnancy, among others [10]. Determinants of birth outcomes, of which maternal anthropometric measurements are a major part, have been demonstrated to differ between developed and developing countries [11–13].

Studies have demonstrated a linear relationship between maternal weight gain and birth weight. Thus a weight gain is popularly associated with normal birth weight whereas a maternal weight loss is associated with low birth weight, among others [14]. Some studies have been conducted into the relationship between maternal anthropometric measurements, other maternal factors and birth weight [11, 14–17].

In the light of the afore-stated, this cross-sectional study was designed to evaluate the associations, if any, between the effect of maternal anthropometric measurements, other maternal factors of expectant women and birth weight of neonates, and to also assess the effect of these same factors on the birth weight of the neonates.

## Methods

### Selection of area, hospitals and subjects

Korle-Bu Teaching Hospital is a flagship urban teaching hospital that serves Accra and other nearby towns and villages. It is the largest hospital in the country with obstetric care at all levels. It registers approximately 10,000 pregnancies annually. It receives referrals from polyclinics and other maternity centers within Accra and surrounding towns and villages and indeed throughout the country.

Ridge Hospital serves as a regional medical centre. Catchment area extends to Winneba, Kasoa, Nsawam, including neighbourhood polyclinics in Accra. There is an open door policy, no cases are turned away.

Women with singleton pregnancies comprised the study population. Nine-hundred and sixty-six singleton pregnant women were randomly sampled in two health facilities (Ridge Hospital, the Greater Accra Regional Hospital, and Korle-Bu Teaching Hospital, a referral facility) in the Greater Accra Region with singleton pregnancies above 28 weeks at delivery and at attendance of antenatal clinics were studied. Systematic Random Sampling, specifically, was employed in this regard. Knowing, on average, the number of women within our inclusion criteria who visited each facility per day, on a daily basis, we picked a random start and thereafter, we picked successive participants, based on the sampling interval till they were all been sampled for the day. Mothers with twin or multiple births, as well as those seriously sick or had physical incapacitation, were excluded from the study.

#### Ethical clearance:

Ethical review was granted by the Ethical Review Committee of the Ghana Health Services. Participants provided written informed consent to share data and participation was completely voluntary.

### Questionnaire

Information about the maternal, demographic and anthropometric measurements were recorded on fully structured questionnaire. The questionnaire was first pre-tested, in a facility different from the two study-facilities, to ascertain the validity of questions enshrined in it and its potential to capture required information for the study adequately well. Questions were finally modified to reflect language simplicity, clarity and ease in comprehension.

### Anthropometric measurements

#### Weight

Weight was taken with the Seca scale. The scale was calibrated each time it was moved. A participant stood still in the middle of the scale’s platform without touching anything and with the body weight equally distributed on both feet. The weight was read to the nearest 100 g (0.1 kg) and recorded immediately (two measurements taken in immediate succession should agree to within 0.1 kg).

#### Height

Each participant was bare footed with heels together, arms to the sides, legs straight, shoulders relaxed. Heels, buttocks, scapulae (shoulder blades), and back of the head were against the vertical board of the stadiometer. The measurement was read to the nearest 0.1 cm and the eye level with the headboard to avoid errors. Since there is no data on pre-pregnancy weight, first registration weight was used. For maternal height measurement reference, 145 cm was used as cut-off for short stature, which is considered a risk criteria for birth weight.

#### Neonate information

Neonatal measurements were obtained within 72 h of delivery. Birth weight was obtained using a calibrated electronic scale. Length was measured using a standardized plastic length board. The newborns were weighed on a paediatric Seca scale that was accurate to within 10 g by research nurses. Adjustments were made to zero for any cushion, a towel or diaper that was in place. The weight of the cushion was subtracted from the newborn’s weight. Infants were weighed nude or with minimum clothing. The average of two weighing was recorded in the infant’s record to the nearest 10 g (0.01 kg). Weight at delivery was used to determine birth weight >90th percentile. Weight at the anthropometric assessment was used to determine total and percent body fat . Sex of the neonate was also established.

#### Blood pressure

After at least 10 min rest, after a woman walked in, blood pressure was measured on two occasions with a mercury sphygmomanometer with an interval of 5 min between measurements with a pressure monitor. Two readings were taken–the “systolic” pressure was recorded as the heart beats, and the second “diastolic” reading was taken during the “rest” between beats. The mean of the last two measurements was used. If BP was above 140/90 on at least two occasions within a week, a diagnosis of elevated blood pressure was made. This was followed by a referral to a competent professional for further evaluation.

#### Hemoglobin testing

A separate consent statement was read to the eligible participants. This statement explained the purpose of the test and requested permission for the test to be carried out. It required just a small sample of easily obtained blood. Before the blood was taken, the finger was wiped with an alcohol prep swab and allowed to air-dry. Then the palm side of the end of a finger was pricked with a sterile, non-reusable, self-retractable lancet and a drop of blood collected. It measured the concentration of hemoglobin in the blood. The results were recorded in the maternal health book, and the book was returned to each mother as per hospital policy. For each participant whose hemoglobin level was lower than the cut-off point of 11 g/dl a referral was made for further evaluation. It is normal for hemoglobin levels to go down somewhat in the second half of pregnancy, when the amount of blood in the body is expanding dramatically. Follow-up hemoglobin (Hb) tests were given in late second trimester or early third trimester for monitoring. Consent was given for blood work.

### Data management and statistical analyses

The questionnaire was reviewed for completeness and consistency before data was entered. To ensure that sample selection procedures were complied with, the author worked closely with the field supervisors and interviewers throughout the data collection period. Effective management of fieldwork was emphasized. Participants were screened based on inclusion criteria and sampling procedures were adhered to. The study questionnaire was pretested to provide face validity in wording and interpretation.

The general characteristics of the study population were examined using mean and standard deviation for continuous variables and frequencies for categorical variables. Cross tabulations were used to establish the relationship between two or more categorical variables. Bivariate analysis was performed by cross tabulation to determine the predictors associated with birth weight which was coded as 1 = normal, 0 = low. Wald statistics was used to test the significance of individual coefficients in the model.

*P*-values obtained through both chi-square analysis and binary logistic regression analysis was employed to assess any associations between BW and maternal demographic and anthropometric variables. These predictor variables in the bivariate model included sex of baby, blood pressure status, meal intake frequency, hemoglobin concentration, gestational age at the time of birth urine protein levels, age of the respondent, employment status, marital status and antenatal care visits. The basis for building the multivariate model was using the Enter approach, in SPSS. Gestational age, in weeks, was calculated from the first day of the last menstrual period (LMP), as ultrasound and sonogram systems were not available. Significance level was defined as *p* < 0.05. The strength of these associations was established using odds ratio and their 95 % Confidence Intervals (CI). Statistical analysis was performed using SPSS software version 20.0 (IBM Corporation, 2011) [18].

## Results and discussion

### Socio-demographic characteristics of respondents

Almost all of the respondents were on health insurance (99.1 %). Over half of the respondents (56.3 %) earned less than GH¢100.00 per month while only 2.5 % earned GH¢1000.00 or more, Table 1. Their occupations were grouped into ‘Professional’, referring to Teachers, Engineers, Medical Doctors, etc., followed by ‘Middle Level Officers’, referring to respondents engaged in white-collar jobs (whether private or public) such as secretaries, managers, etc. The rest were ‘Trades’, referring to artisans such as hair-dressers, seamstresses, caterers, etc., and finally traders, also referring to traders in the local markets or business women operating mini-markets or super markets, etc. It turned out that less than half of the respondents (34.5 %) were traders, 5.5 % were professionals, while 4.6 % were middle level officers. Almost 40 % of them were unemployed. The modal parity was 1 and the median was 2. Parity was however re-grouped into 0–1, 2–3 and ≥4. About 44 % were expecting up to their second child while a little over 11 % were expecting at least their fourth child. A great majority of them were married (85.4 %) while a little over half of them had completed either Junior Secondary or Senior Secondary school. About 9.6 % had either never been to school or had completed primary school, and 35.9 % had completed tertiary education. The ages of the respondents were pre-grouped from ‘Under 20 years’,’20–29 years’ all the way to’40 years and above’. The modal age group was 20–29 year (48.8 %) closely followed by 30–39 years (43.1 %) whereas the least was 40 years and above (3.2 %), Table 1.

**Table 1:** Socio-demographic characteristics of respondents

Variables and levels	N (%)
Health insurance status	
Yes	957 (99.1)
No	9 (0.9)
Average monthly income (in GHC)	
<100	378 (56.3)
100–499.9	211 (31.4)
500–999.9	66 (9.8)
≥1000	17 (2.5)
Occupation	
Professional	53 (5.5)
Middle level officer	44 (4.6)
Trades	245 (25.5)
Trader	331 (34.5)
Unemployed	287 (39.9)
Parity	
0–1	422 (43.7)
2–3	436 (45.1)
≥4	108 (11.2)
Marital status	
Single	141 (14.6)
Married	825 (85.4)
Education	
Primary	93 (9.6)
Secondary	526 (54.5)
Tertiary	347 (35.9)
Age (years)	
<20	48 (5.0)
20–29	471 (48.8)
30–39	416 (43.1)
≥40	31 (3.2)

Exchange rate:1US$ = 3GHC

### Anthropometric measurements, gestation and hemoglobin levels of women at birth

Table 2 contains the frequencies of gestation at birth, systolic and diastolic blood pressure, hemoglobin at birth, and BMI at birth. There is a significant positive correlation between systolic blood pressure and baby’s weight at birth (*r* = 0.216, *p* = 0.001), diastolic blood pressure and baby’s weight at birth (*r* = 0.214, *p* = 0.001), and parity and baby’s weight at birth (*r* = 0.140, *p* = 0.021) and between mother’s age and baby’s weight at birth (0.108, *p* = 0.05). The mean gestation at birth was almost 39 weeks, the mean BMI of mother at birth was 28.8 kg/m^2^, and the mean systolic blood pressure at birth was 114.5 mmHg while that of the diastolic blood pressure at birth was 70.3 mmHg. The hemoglobin at birth was 11.3. The anthropometric measurements were re-grouped in Table 2. Majority (over 60 %) of the mothers were in the 35–39.9 gestation at birth group while a similar proportion (over 60 %) of the mothers were in the 90–119.9 mmHg group. Majority of the mothers (over 80 %) had their diastolic blood pressure in the 60–89.9 mmHg group. The most represented hemoglobin at birth (25.6 %) fell in the 11–11.9 group. The most common BMI at birth (58.0 %) fell in the 20–29.9 kg/m^2^ group.

**Table 2:** Anthropometric measurements, gestation and hemoglobin levels of women at birth

Parameters and levels	N (%)
Gestation at birth (weeks)	
Less than 30	28 (2.9)
30–34.9	139 (14.4)
35–39.9	589 (61.1)
40 and above	208 (21.6)
Systolic blood pressure at birth (mm Hg)	
Less than 90	8 (0.8)
90–119.9	590 (61.2)
120–149.9	312 (32.4)
150 and above	54 (5.6)
Diastolic blood pressure at birth (mm Hg)	
Less than 60	74 (7.7)
60–89.9	783 (81.2)
90–119.9	97 (10.1)
120 and above	10 (1.0)
Hemoglobin at birth (g/dl)	
Less than 9	67 (7.0)
9–9.9	134 (13.9)
10–10.9	233 (24.2)
11–11.9	246 (25.6)
12–12.9	184 (19.1)
13 and above	98 (10.2)
BMI at birth (kg/m^2^)	
Less than 20.0	42 (4.5)
20.0–29.9	538 (58.0)
30.0–39.9	312 (33.6)
40 and above	36 (3.9)

Whereas majority of the low birth weights were recorded among the female neonates, (60.3 %), the opposite was the case for the recorded normal birth weights (55.2 % of male neonates), Table 3. On birth weight and hemoglobin levels of mothers, whereas majority of both low birth weights and normal birth weights were non-anemic, the proportion of the anemic among the low birth weights (33.8 %) was almost twice that recorded among the normal birth weights (19.6 %). It is also interesting to quickly point out that whereas majority (64.5 %) of the low birth weights were premature births, the opposite was the case among the normal birth weights (71.8 %).

**Table 3:** Prevalence of low birth weight and normal birth weight

		Weight of baby	
		Low birth weight (Less than 2.5)	Normal birth weight (2.5 and above)
		N (%)	N (%)
Sex of baby	Male	29 (39.7)	481 (55.2)
	Female	44 (60.3)	390 (44.8)
HB status of mother	Anaemic	24 (33.8)	168 (19.6)
	Non-anaemic	47 (66.2)	690 (80.4)
Gestation status	Pre-mature	40 (64.5)	213 (28.2)
	Normal	22 (35.5)	542 (71.8)

In Table 4, there is confirmation to the finding in Table 3 that the low birth weights recorded a significantly (*p* < 0.001) lower gestation (34.8 ± 3.8 weeks) than the normal birth weights (37.3 ± 3.3). It is also the case, per the underlying data, that mothers of normal weight neonates (29.0 ± 6.3) have a significantly (*p* = 0.034) higher BMI than their low birth weight counterparts (27.3 ± 5.4). However, the systolic blood pressure of mothers of normal birth weight neonates (113.3 ± 17.0) are significantly lower (*p* < 0.001) than that of the mothers of low birth weight neonates (122.2 ± 30.0). A similar picture can be seen with the case of diastolic blood pressure (69.8 ± 13.2 for normal birth weight and 75.6 ± 20.7 for low birth weight with a significance of *p* = 0.001). However, mothers’ hemoglobin levels had no significant association with birth weights of neonates, Table 4.

**Table 4:** Association between birth weight of neonates and anthropometric characteristics of mothers

Parameters	LBW	NBW	*t*-test
Gestation at birth (weeks)	34.8 ± 3.8	37.3 ± 3.3	<0.001
BMI at birth (kg/m^2^)	27.3 ± 5.4	29.0 ± 6.3	0.034
SBP (mm Hg)	122.2 ± 30.0	113.3 ± 17.0	<0.001
DBP (mm Hg)	75.6 ± 20.7	69.8 ± 13.2	0.001
HB (g/dl)	11.6 ± 8.4	11.2 ± 3.4	0.39

One of the areas of interest in this study was how the mean birth weights of the neonates were affected by the various levels of the socio-demographic and anthropometric parameters of mothers, Table 5. As the BMI of the mother generally rises, the birth weight of her neonate also rises, however this association is not statistically significant at 95 % confidence level, Table 5. This same trend show in the remaining parameters investigated such as Age of mother, Educational level of mother, Mother’s occupation, Mother’s monthly income, Gestation at birth, Systolic and Diastolic blood pressures and Mother’s haemoglobin level at birth were seen to have no significant association with the birth weight of the neonates.

**Table 5:** Association between socio-demographic and anthropometric characteristics of mothers and birth weights of neonates

Parameter	Level	Number	Mean weight of baby (kg)	F (p)
Age of mother (years)	<20	46	3.001	0.975 (p>0.05) (NS)
	20–29	464	3.257	
	30–39	406	3.303	
	≥40	30	3.445	
Education of mother	Primary	89	3.185	0.225 (p>0.05) (NS)
	Secondary	517	3.283	
	Tertiary	340	3.273	
Occupation	Professional	53	3.323	0.088 (p>0.05) (NS)
	Middle level officer	43	3.303	
	Trades	241	3.246	
	Trader	320	3.272	
	Unemployed	289	3.279	
Monthly income (GHc)	<100	369	3.261	0.119 (p>0.05) (NS)
	100–500	206	3.261	
	500–1000	64	3.35	
	>1000	17	3.276	
Gestation at birth (weeks)	Less than 30	28	3.013	1.359 (p>0.05) (NS)
	30–34.9	137	3.12	
	35–39.9	576	3.289	
	40 and above	204	3.356	
Systolic blood pressure (mm Hg)	Less than 90	8	2.838	1.505 (p>0.05) (NS)
	90–119.9	579	3.25	
	120–149.9	304	3.363	
	150 and above	54	3.034	
Diastolic blood pressure (mm Hg)	Less than 60	73	3.124	0.383 (p>0.05) (NS)
	60–89.9	769	3.279	
	90–119.9	93	3.322	
	120 and above	10	3.23	
Haemoglobin at birth (g/dl)	Less than 9	66	3.013	0.606 (p>0.05) (NS)
	9–9.9	130	3.322	
	10–10.9	230	3.283	
	11–11.9	243	3.295	
	12–12.9	178	3.264	
	13 and above	96	3.295	
Body mass index (kg/m^2^)	Less than 20.0	41	3.227	0.726 (p>0.05) (NS)
	20.0–29.9	529	3.226	
	30.0–39.9	306	3.349	
	40.0 and above	34	3.409	

Exchange rate:1$ = 3GHC

* p < 0.05; *NS* Not significant

For the binary logistic regression, the Nagelkerke R Square (=0.236) shows that about 23.6 % of the variation in the outcome variable (Birth Weight) is explained by this logistic model. In the Wald statistics (this determines the relative importance of the predictor variables in predicting the response, thus the higher the more important and vice versa) column of Table 6, Gestation-at-birth (Wald = 9.4) happens to be the most important predictor of a mother’s likelihood of having normal birth weight. This is followed by Diastolic-blood-pressure-at-birth (Wald = 7.08), and mother’s-weight-at-birth (Wald = 7.07). However the remaining variables were not significant in predicting a mother’s likelihood of having normal birth weight baby.

**Table 6:** Results of binary logit - determinants of birth weight

	B	S.E.	Wald	Sig.	Exp(B)	95 % C.I. for Exp(B)	
						Lower	Upper
Age (years)	−0.046	0.276	0.028	0.868	0.955	0.557	1.64
Education	−0.3	0.172	3.048	0.081	0.741	0.529	1.037
Occupation	−0.466	0.247	3.57	0.059	0.628	0.387	1.018
Monthly income (GHc)	.013	.274	.002	0.961	1.013	.592	1.733
Haemoglobin at birth (g/dl)	.286	.446	.411	0.521	1.331	.555	3.189
Mothers BMI (kg/m^2^)	.050	.030	2.688	0.101	1.051	.990	1.115
SBP at birth (mm/Hg)	−.010	.012	.780	0.377	.990	.967	1.013
DBP at birth (mm/Hg)	−.048	.018	7.069	0.008	.953	.921	.988
Gestation at birth (weeks)	−.708	.382	3.426	0.064	.493	.233	1.043

Diastolic-blood-pressure-at-birth (0.008) was the only significant factor that predicted baby’s weight at birth. The Exp (B) and 95 % C.I. columns of Table 6 give us the odds ratios and their corresponding 95 % confidence interval estimates respectively. An increase in a mother’s diastolic blood pressure at birth has a 4.7 % (95 % CI 2.2 to 7.9 %) decrease in the odds of having a normal birth weight baby.

The overall accuracy of this model to predict subjects having normal birth weight (with a predicted probability of 0.5 or greater) is 93.7 % (Table 6). The sensitivity is given as 497/498 = 99.8 % and the specificity is 6/39 = 15.4 %. The model has a positive predictive value (PPV) = 497/530 = 93.8 % and negative predictive value (NPV) = 6/7 = 85.7 %.

### Discussion

The bulk of incidents of low birth weight are found in developing countries, the major cause of which is preterm birth (<37 weeks) [15]. One key finding in this study is the fact that the mothers of neonates with low birth weight had significantly lower BMI and gestation at birth than their normal birth weight counterparts. This is seen in the association between birth weight and anthropometric characteristics of mothers in this article. We could not however demonstrate, with the study data, that maternal characteristics such as age, education, occupation, monthly income and haemoglobin at birth are significant determinants of birth weight of neonates. This is unlike studies that have demonstrated that poor families are more likely to have LBW neonates than well to do ones [19]. In their study titled “Maternal anthropometric measurements and other factors: relation with birth weight of neonates”, Tabrizi and Saraswathi [20] considered family income, among others, as a predictive factor of birth weight of neonates [21].

Meanwhile, several studies across the world have shown that, education affects neonatal birth weight [6, 19, 21], though it has been established in literature that the causes of LBW in neonates differ between developing and developed societies [15, 22–25]. It is also reported in de AlencarBritto et al. that age, BMI and family income were significantly associated with LBW in neonates [26]. However in this study, they were not.

There was no significant association between gestation at birth and birth weight of neonates, mother’s BMI and birth weight of neonates, systolic blood pressure and birth weight of neonates, as well as diastolic blood pressure and birth weight of neonates. Though the gestation at birth, mothers’ height and weight at birth were lower for LBW neonates than their NBW counterparts, that difference was not statistically significant. Whereas the systolic and diastolic blood pressures and were higher for mothers of LBW neonates than their NBW counterparts, only the diastolic blood pressure showed a statistically significant difference. These findings in the current study run through several studies over the world [20, 27, 28]. However, in developing countries, gestation at birth is a determinant of birth weight [29].

There was a significant positive association between the BMI of mothers, on one side, and the weights of their neonates. Thus mothers of normal birth weight neonates were also significantly taller and heavier than their low birth weight counterparts [29]. This is similar to the findings of Momen et al. [17] in their study titled *Anthropometric assessment of nutritional status of Bangladeshi pregnant women and weight of their newborns*, conducted in Bangladesh. In their study they found a positive association between the weight of mothers and that of their neonates. Similarly, these findings in the current study also run through the studies of Hassan et al., titled *relationship between maternal characteristics and neonatal birth size in Egypt,* in which correlation tests between maternal and neonatal anthropometric measurements revealed that for both sexes combined maternal weight had a significant positive correlation with neonatal weight [27]. The relationship between the BMIs of mothers and the birth weights of their neonates was not statistically significant in this study as a Bangladeshi study revealed that a combination of the initial weight and height of the mother was not a good determinant of neonatal birth weight [27]. The same study showed that maternal weight was the best determinant of neonatal birth weight whereas in this current study, gestation at birth proved to be the best determinant of neonatal birth weight. However, other studies have also shown that not only are BMI, weight and height predictors of birth weight of neonates but that the most important predictor of birth weight is weight at first visit, followed by BMI and then Height, in descending order [29]. On the other hand, this study found that Gestation-at-birth was the most important determinant of birth weight of neonates, followed by Diastolic Blood Pressure and mother’s weight-at-birth for the study area.

Mothers’ hemoglobin levels had no association with birth weights of their neonates in this current study. Other studies have however shown the contrary [30–32].

## Strengths and limitations

In several other studies, factors such as birth order, antenatal care and mother’s health status were found to also be significant predictors of birth weight of neonates [22, 23–35]. However, in this study we did not consider those factors together with birth order in the prediction of the birth weight of neonates. These could be a limitation with our study. Furthermore, given that the Korle-Bu teaching hospital is generally a referral hospital, and the fact that it contributed a larger portion of respondents, may suggest that factors discovered as being associated with birth weight may have been over-represented in this study. Another limitation could be the inability of this study to capture the quality of education, as well as the subjective nature in which the education and monthly income variables were captured. These limitations notwithstanding, the results of this current study are considered representative given the sampling technique. The strengths of this current study are a large sample size, collection of birth data from the cohorts of mothers studied by well-trained research staff and the fact that birth data of the neonates were obtained from reliable standard instruments, rather than recall.

## Conclusions

It is evident from this study that among mothers’ characteristics, Diastolic Blood Pressure was the only important determinant of birth weight of neonates. Mothers’ Systolic Blood Pressure, Haemoglobin at birth and mother’s height were not significant per the binary logistics regression. However among the socio-demographic characteristics of mothers, Age of mother, level of education, occupation and monthly income were not significant in determining the birth-weight of neonates’.

Mothers of neonates with low birth weight were found to have significantly higher systolic and diastolic blood pressures, lower weight and shorter in terms of height as well as lower gestation at birth than their normal birth-weight counterparts. There was no significant difference between the haemoglobin level of mothers of normal birth weight and their low birth weight counterparts.

### Ethical clearance

Ethical review was granted by the Ethical Review Committee of the Ghana Health Services. Informed written consent was requested from study participants. The participation by any person in the study was voluntary. A separate consent was obtained for blood work including hemoglobin. They were informed that upon completion of the work the results would be shared with them and the community. This will help inform interventions on maternal child health services.

## Abbreviations

ANC: Antenatal care
IUGR: Intra uterine growth retardation
LBW: Low birth weight
LMP: Last menstrual period
MCH: Maternal child health
MOH: Ministry of health
NBW: Normal birth weight
UN: United Nations
UNICEF: United Nations Children Education Fund
WH: World Health Organization
KBTH: Korle-Bu Teaching Hospital
